# Normal Development in Mice Over-Expressing the Intracellular Domain of DLL1 Argues against Reverse Signaling by DLL1 In Vivo

**DOI:** 10.1371/journal.pone.0079050

**Published:** 2013-10-22

**Authors:** Christian Redeker, Karin Schuster-Gossler, Elisabeth Kremmer, Achim Gossler

**Affiliations:** 1 Institut für Molekularbiologie OE5250, Medizinische Hochschule Hannover, Hannover, Germany; 2 Institut für Molekulare Immunologie, Helmholtz Zentrum München, Forschungszentrum für Umwelt und Gesundheit, GmbH, München, Germany; Instituto de Medicina Molecular, Portugal

## Abstract

The Notch signaling pathway mediates the direct communication between adjacent cells and regulates multiple developmental processes. Interaction of the Notch receptor with its ligands induces the liberation of the intracellular portion of Notch (NICD) referred to as regulated intramembraneous proteolysis (RIP). NICD translocates to the nucleus, and by complexing with the DNA binding protein RBPjκ and other cofactors activates transcription of bHLH genes. RIP-like processing of various mammalian Notch ligands (DLL1, JAG1 and JAG2) and the translocation of their intracellular domains (ICDs) to the nucleus has also been observed. These observations together with effects of over-expressed ligand ICDs in cultured cells on cell proliferation, differentiation, and Notch activity and target gene expression have led to the idea that the intracellular domains of Notch ligands have signaling functions. To test this hypothesis in vivo we have generated ES cells and transgenic mice that constitutively express various versions of the intracellular domain of mouse DLL1. In contrast to other cell lines, expression of DICDs in ES cells did not block proliferation or stimulate neuronal differentiation. Embryos with ubiquitous DICD expression developed to term without any apparent phenotype and grew up to viable and fertile adults. Early Notch-dependent processes or expression of selected Notch target genes were unaltered in transgenic embryos. In addition, we show that mouse DICD enters the nucleus inefficiently. Collectively, our results argue against a signaling activity of the intracellular domain of DLL1 in mouse embryos in vivo.

## Introduction

The Notch pathway is a highly conserved signaling mechanism that mediates local interactions between adjacent cells. Notch signaling is pivotal for the regulation of developmental processes in a wide variety of different tissues and species [reviewed in 1-7]. The Notch gene of Drosophila as well as its vertebrate homologues encode large transmembrane proteins with numerous EGF-like repeats in their extracellular domains. On the cell surface these receptors interact with products of the Delta, and Serrate (called Jagged in vertebrates) genes that also encode transmembrane proteins with a variable number of EGF-like repeats in their extracellular domains [8-10]. The Notch protein is proteolytically processed in the Golgi network and reaches the cell surface as a non-covalently linked heterodimeric receptor [11,12]. Upon ligand binding, the intracellular portion of Notch (NICD) is liberated by two successive proteolytical cleavages by sheddases and γ-secretase referred to as regulated intramembraneous proteolysis (RIP). NICD translocates to the nucleus, and by complexing with the transcriptional regulator RBPjκ and other cofactors activates transcription of bHLH genes [13-19]. Their gene products in turn regulate the transcription of other downstream effector genes. 

RIP-like processing in cultured cells has also been reported for the Notch ligands Drosophila Delta, for vertebrate Delta1, and Jagged 1 and 2 [20-24]. Cleavage products corresponding to the intracellular domains of endogenous rat Jagged1 or over-expressed Xenopus Delta(DL)1 and Serrate (Jagged) 1 were detected in lysates of rat and Xenopus embryos, respectively [23,24]. Similarly, cleavage products corresponding to the intracellular domains of endogenous mouse DLL1 was detected in isolated neuronal stem cells of E10.5 embryos [25], indicating that RIP of DSL proteins occurs in various vertebrate species in vivo. The released intracellular domains (ICDs), except Xenopus DL1, were detected in the nucleus at varying levels [20-24,26], leading to the suggestion that the ICDs have a nuclear function and may mediate bidirectional or reverse signaling of the Notch pathway. Indeed, overexpression of the ICD of Xenopus Serrate (Jagged) 1 in Xenopus embryos inhibited primary neurogenesis [24], and in HUVEC and NIH3T3 cells overexpression of human DL1ICD caused cell cycle arrest through up-regulation of the cell cycle inhibitor p21. This effect did not depend on nuclear localization and was antagonized by concomitant overexpression of NICD suggesting antagonistic activities of DL1ICD and NICD[27]. Furthermore, mouse DL1ICD stimulated neuronal differentiation in isolated embryonic neural stem cells and induced neuronal differentiation in P19 embryonal carcinoma cells through binding to Smad proteins and stimulation of TGF-β signaling [25]. However, in frog embryos over-expressed Xenopus DL1ICD had no effect on neurogenesis [24]. In HEK293 cells mouse DL1ICD suppressed NICD-induced target gene expression by preventing the physical interaction of NICD and RBPjκ, suggesting that cleaved DLL1 can antagonize Notch signaling in DLL1 expressing cells [26]. Collectively these findings raise the possibility of signaling roles for DL1ICD in various cellular contexts and an attenuation of Notch signaling by DL1ICD. However, whether DL1ICD has signaling activity and stimulates differentiation and/or antagonizes Notch signaling during development under physiological conditions in vivo is unclear. 

Here, we report on the generation and analysis of mice that over-express various mouse DL1ICD constructs under the control of the CAG promoter. Despite ubiquitous expression at higher levels than endogenous DLL1 we did not observe developmental defects or changes in gene expression that would indicate an inhibitory effect on Notch signaling or any other significant role of the mouse DL1ICD during embryogenesis. Thus, a signaling function of the intracellular domain of DLL1 in mouse embryos in vivo appears unlikely.

## Results and Discussion

### Generation of ES cells allowing for Cre-mediated activation of DL1ICD expression

**Figure 1:**
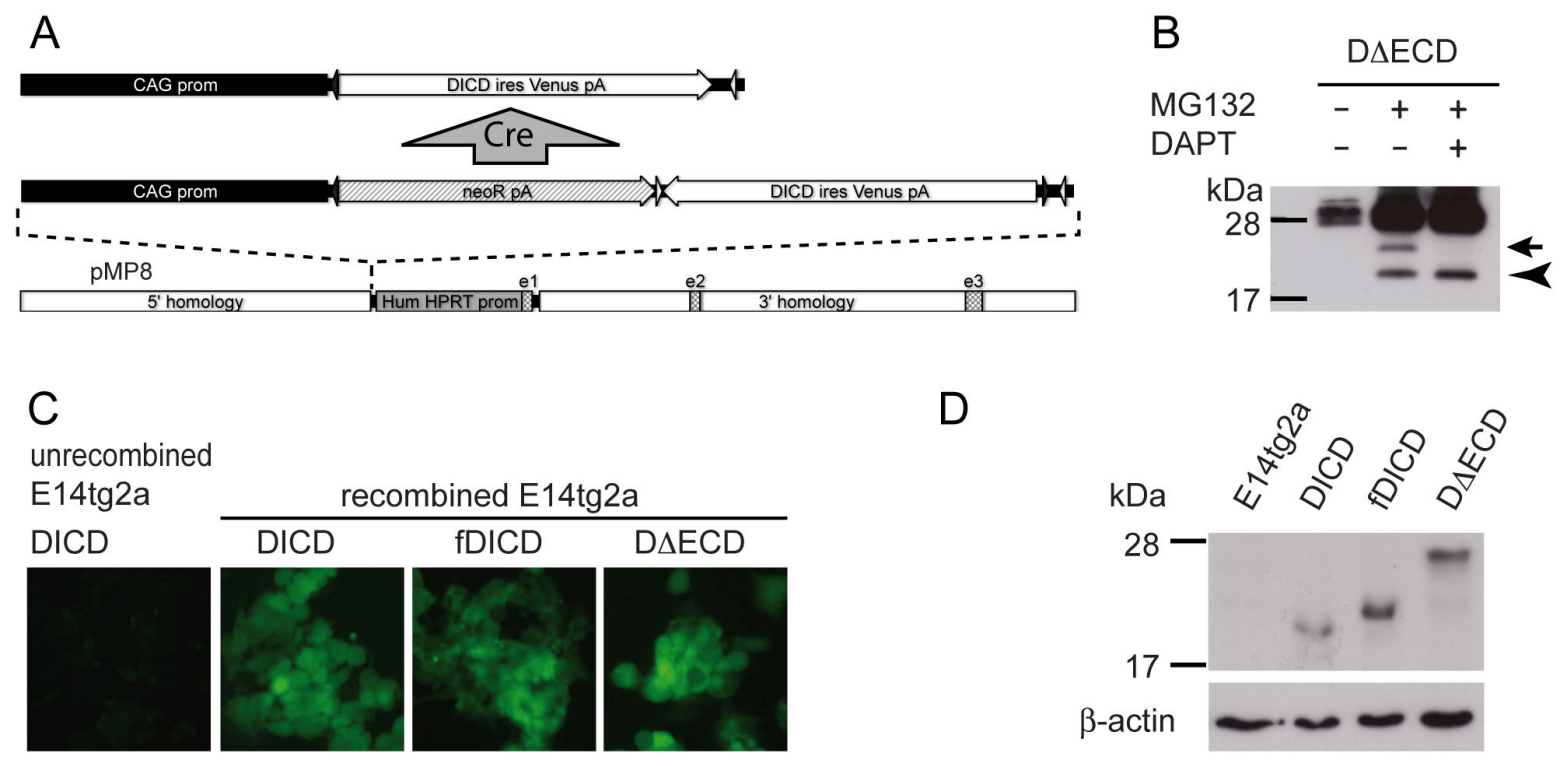
Inducible expression of DL1ICD variants in ES cells. (A) Schematic representations of the expression construct prior to and after Cre-mediated recombination, and of the pMP8 targeting vector. Black and white triangles indicate wild type loxP and mutant loxP2272 sites, respectively. (B) Western blot analysis of HA-tagged DΔECD expressed in CHO cells showing in addition to a γ-secretase-dependent cleavage product (arrow) a γ-secretase-independent proteolytic fragment (arrow head). (C) GFP expression in targeted ES cells indicating Cre-mediated activation of transgene expression. (D) Western blot analysis of cell lysates from wild type and DL1ICD-expressing E14tg2a cells using affinity purified polyclonal anti-DICD peptide antibodies.

If DL1ICD has signaling functions and/or interferes with Notch signaling in vivo constitutive or widespread overexpression of DL1ICD might prevent the generation of living transgenic mice as is the case with overexpression of NICD [28]. To circumvent this potential problem we used a strategy to conditionally activate CAG promoter-driven expression of mouse DL1ICD transgenes inserted into the HPRT locus of ES cells, and in ES cell-derived transgenic mice. In these constructs the CAG promoter is upstream of a stop cassette, which is flanked by a wild type loxP site at the 5' end and a mutant loxP2272 site at the 3' end. Downstream of the stop cassette resides the transgene of interest in reverse orientation preventing leaky transgene expression. The transgene is followed by a wild type and mutant loxP site, which both are in opposite orientation to the corresponding sites flanking the stop cassette. Upon recombination with Cre the stop cassette is excised and the orientation of the transgene corrected (Figure 1 A; [29]). Since tagged and untagged versions of DL1ICD were used in previous in vitro studies, for better comparison and to exclude potential effects of the tag, we generated expression constructs for untagged DL1ICD (from hereon referred to as DICD), and N-terminally flag-tagged DL1ICD (from hereon referred to as fDICD). Furthermore, we generated a version of DLL1 with a deletion of the extracellular domain (from hereon referred to as DΔECD), whose intracellular domain is released by γ-secretase (arrow in Figure 1 B), in addition to another γ-secretase-independent cleavage that generates a smaller C-terminal fragment (arrowhead in Figure 1 B). DΔECD was included because proteolytic and potentially other (unknown) processing events at the membrane might influence DICD activity in vivo. In all constructs the DL1ICD coding sequence was linked to IRES Venus. Inducible DL1ICD expression constructs were cloned into the HPRT targeting vector pMP8 (Figure 1 A)[30,31] and introduced by homologous recombination into E14TG2a ES cells, which carry a deletion at the HPRT locus that renders HPRT inactive [32]. Homologous recombination with pMP8 restores HPRT activity and renders E14TG2a cells HAT resistant, which allows for direct selection of single copy insertions of DL1ICD expression constructs in ES cells. 

### DL1ICD expression in ES cells has no significant effect on proliferation or neuronal differentiation

To analyze the effects of DL1ICDs in ES cells clonal lines carrying the respective recombined targeting vectors were established indicated by expression of Venus (Figure 1 C). Expression of the respective DL1ICD variants was analyzed by Western blotting (Figure 1 D) using a polyclonal antibody that is directed against a peptide of the DLL1 intracellular domain. Expression of all DL1ICDs was detected but steady state levels of DL1ICD proteins differed, DICD being the least abundant protein, which most likely reflects different stabilities of the DL1ICD variants. No endogenous DLL1 protein was detected, although Dll1 transcripts have been found in ES cells by RT-PCR [33]. Thus, CAG promoter-driven expression of single copy DL1ICD transgene insertions in the HPRT locus resulted in readily detectable expression of DL1ICD proteins that exceed endogenous levels. 

**Figure 2:**
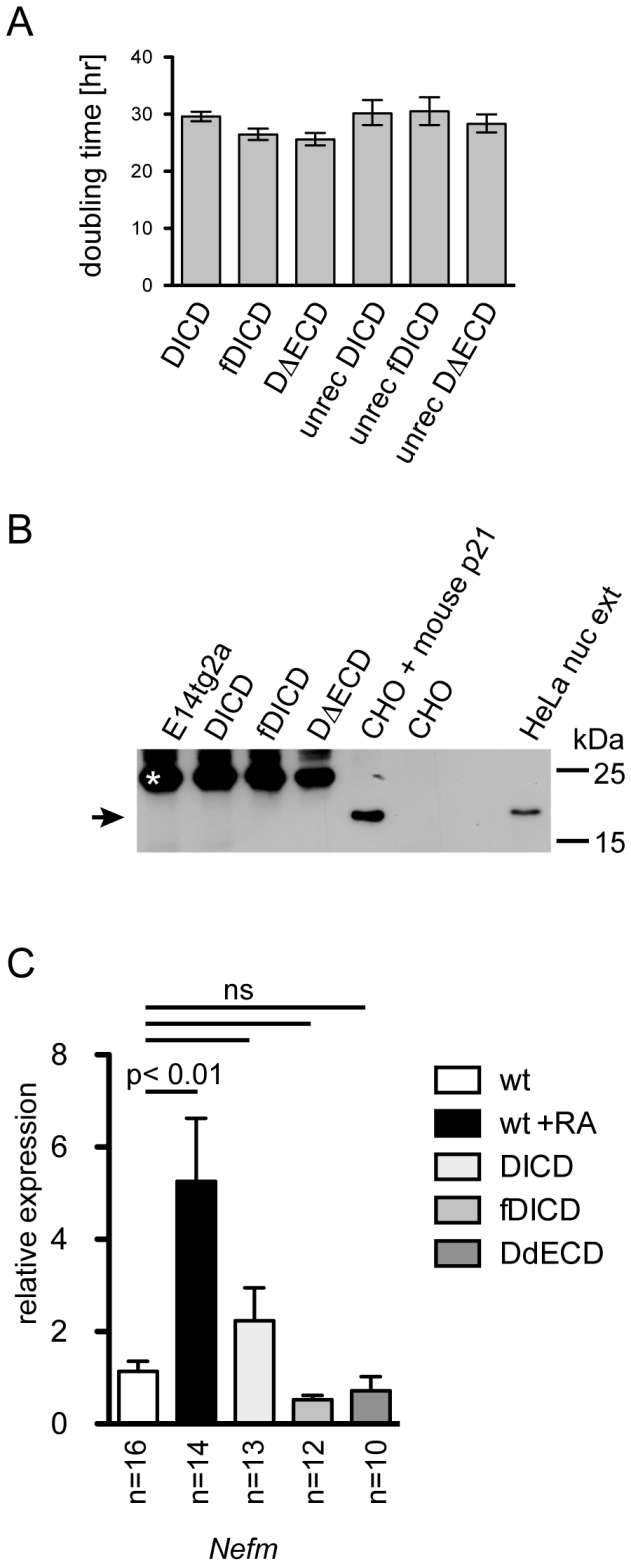
Normal proliferation and neuronal differentiation of ES cells expressing DL1ICD variants. (A) Doubling times of targeted E14tg2a cells before and after Cre-mediated activation of DL1ICD expression. Doubling times were calculated from cell counts after non-linear regression using Prism software (GraphPad). Indicated are mean doubling times and upper and lower limit of 95% confidence intervals. (B) Western blot analysis of cell lysates of wild type and DL1ICD-expressing ES cells, CHO cells with or without transient expression of mouse p21, and HeLa nuclear extract. The arrow points to the position of p21, the asterisk marks a non-specific background band detected in ES cells. (C) Expression of the pan-neuronal marker Nefm in differentiated wild type and DL1ICD-expressing ES cells analyzed by qRT-PCR. Indicated are means and SEM of expression levels determined in differentiated wild type (n=16 pools of aggregates ) RA treated (n=14 pools of aggregates) and transgenic (DICD: n=13 pools of aggregates; fDICD: n=12 pools of aggregates; DΔECD: n=10 pools of aggregates) ES cells. ns: not significant (p>0.05).

ES cells expressing DL1ICD proteins could be readily expanded and maintained, with no apparent difference to targeted ES cells carrying unrecombined (inactive) transgenes. Since overexpression of human and mouse DL1ICD in HUVEC, HEK292 and NIH3T3 cells severely reduced cell proliferation [27,34] and caused up-regulation of the cell cycle inhibitor p21[27] we analyzed cell proliferation of and p21 expression in DICD, fDICD and DΔECD expressing ES cells in comparison to ES cell clones prior to recombination. Doubling times of DICD expressing ES cells varied between 25.6 and 29.6 hr compared to 28.1 to 30.5 hr of ES cells carrying the unrecombined constructs (Figure 2 A), indicating apparently normal cell proliferation. This contrasts with the reduction of cell proliferation by 50-90% that was observed previously in HUVEC, HEK292 and NIH3T3 cells [27,34]. As reported earlier [35] p21 levels in non-transgenic ES cells were below the level of detection (Figure 2B). Similarly, no p21 was detected by Western blot analyses in ES cells expressing DL1ICD proteins (Figure 2B). Thus, in contrast to other cells in culture overexpression of DICD variants in ES cells had only a minor effect on cell proliferation and did not increase p21 expression to detectable levels. The previously observed effect of DL1ICD on cell proliferation and p21 levels might either be cell type-dependent, or reflect significantly higher expression levels that might have been obtained by transient or adenoviral overexpression [27,34]. 

To test whether DL1ICD expression in ES cells stimulates their neuronal differentiation as was observed in P19 cells [25], wild type E14TG2a cells and cells expressing the three DL1ICDs were differentiated in vitro, and expression of Nefm as a pan neuronal marker was analyzed by real time RT-PCR as indicator for neuronal differentiation. Retinoic acid (RA) is a well-known inducer of neuronal differentiation of ES cells [36]. Therefore E14TG2a cells treated with retinoic acid (RA) were used as positive control. Addition of RA to embryoid bodies generated with wild type ES cells induced a 5-fold increase of Nefm mRNA compared to untreated ES cells (Figure 2C) indicative of and consistent with enhanced neuronal differentiation. None of the DL1ICD variants led to a significant increase of Nfem transcription (Figure 2C), indicating that the expression of DL1ICD in ES cells does not stimulate neuronal differentiation. Collectively, our results show that DL1ICDs expression in ES cells from the CAG promoter does not elicit effects that have been observed by overexpression of DL1ICD proteins in other cell types. 

### Normal development of mice expressing DICDs

**Figure 3:**
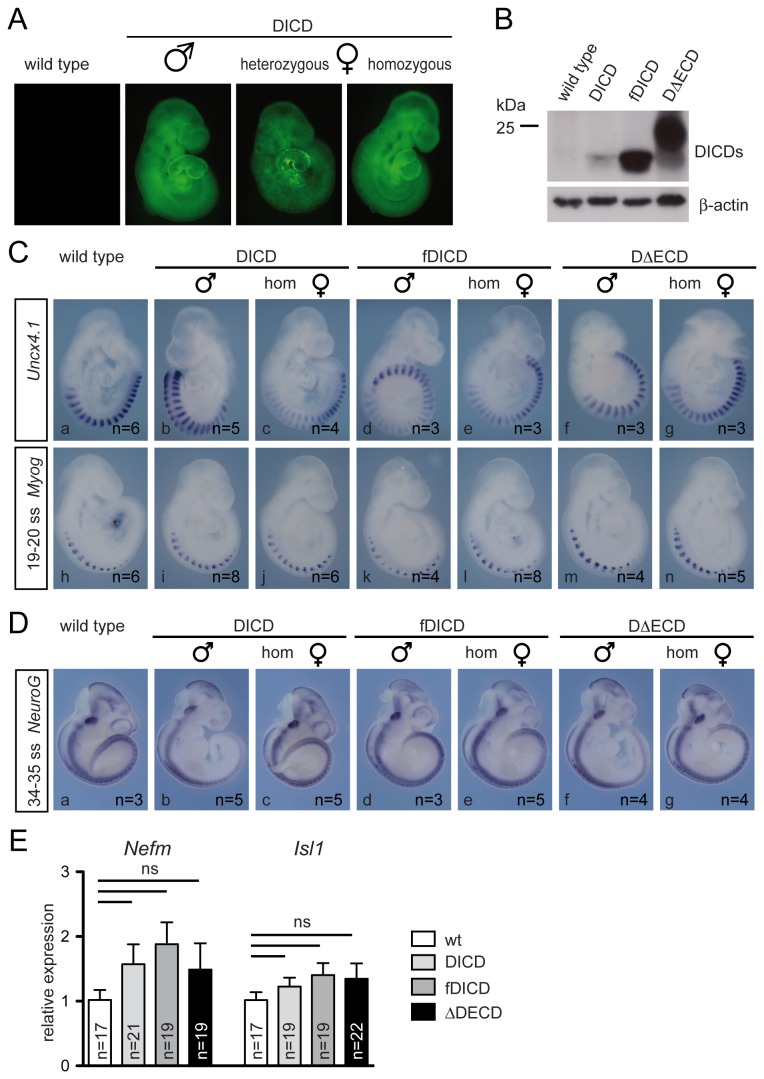
Normal development of embryos expressing DL1ICD variants. (A) GFP expression in male, and hetero-and homozygous female transgenic embryos indicating Cre-mediated activation of transgene expression. (B) Western blot analysis of cell lysates from wild type and DL1ICD-expressing embryos. (C) Whole mount in situ hybridization of wild type (a, h) and DL1ICD-expressing (b-g, and i-n) embryos showing normal anterior-posterior somite patterning (a-g) and muscle differentiation (h-n). (D) Whole mount in situ hybridization of wild type (a) and DL1ICD-expressing (b-g) embryos showing normal neuronal differentiation. (E) qRT-PCR analysis of Nfem and Isl1 expression in wild type and DL1ICD-expressing embryos. Indicated are means and SEM of expression levels determined in individual wild type and transgenic embryos. ns: not significant (p>0.05).

To analyze whether over-expressed DL1ICD affects embryonic development and Notch activity under physiological conditions in vivo E14TG2a cells carrying the unrecombined DICD, fDICD or DΔECD construct, respectively, were used to generate chimeras and transgenic mice. Transgenic mouse lines carrying the DICD and fDICD construct were obtained with two independently targeted E14TG2a cells, and one line carrying the DΔECD construct. Recombination of DL1ICD constructs in embryos was induced by matings of wild type males to females carrying a ZP3Cre transgene that causes site-specific recombination during oogenesis, and one of the DL1ICD constructs, respectively [37], to induce ubiquitous transgene expression. Male embryos derived from these mating carrying a DL1ICD construct expressed Venus ubiquitously whereas female embryos showed mosaic expression due to random X-inactivation (Figure 3A, and data not shown). Similarly, DICD expression detected by whole mount situ hybridization (WISH) using a probe for the intracellular domain was uniform in male embryos (except for the presomitic mesoderm (psm) where stronger staining reflecting the highest levels of endogenous Dll1 mRNA was observed) and patchy in female embryos (data not shown). To ascertain expression of exogenous DL1ICD proteins during development lysates of male transgenic E10.5 embryos were analyzed by Western blotting. All DL1ICD variants were readily detected (Figure 3B). Steady state levels differed consistent with the results obtained from the analysis of DL1ICD expression in ES cells. Importantly, endogenous DICD was below the level of detection in whole embryo lysates under these conditions (Figure 3B), indicating that all exogenous DL1ICD variants were present at higher levels than endogenous DICD. 

Both male and female embryos ubiquitously expressing the DL1ICD variants developed to term showed no externally visible abnormalities, and were fertile. Homozygous female embryos derived from matings of males and females carrying activated DL1ICD constructs expressed Venus indistinguishable from male embryos (Figure 3A), and were also viable and fertile without any apparent phenotype. Because the transgenic lines obtained from independent targeting events behaved virtually identical further analyses were confined to one line, respectively. Female and male transgenic mice were obtained at Mendelian ratios (Table 1), indicating normal development of transgenic embryos expressing any of the DL1ICD variants, and suggesting that none of these DL1ICD proteins has a major impact on embryonic development and Notch pathway function in vivo. 

**Table 1:** Number of offspring with different genotypes obtained from matings of heterozygous transgenic females with transgenic males.

	Genotype				
Construct	X^ICD^/X^ICD^	X^ICD^/X	X^ICD^/Y	X/Y	Total
DICD	13	21	21	11	66
fDICD	9	15	14	12	50
DΔECD	15	12	15	9	51

X^ICD^ and X indicate transgene-carrying or wild type X chromosomes, Y the Y chromosome. The obtained numbers of offspring were analyzed by Chi Square test and did not differ significantly from expected values.

To detect potential subtle effects caused by DL1ICD overexpression during development we concentrated on anterior-posterior somite patterning, and muscle and neuronal differentiation, processes known to require DLL1 and Notch signaling [28,38-41]. To detect abnormalities in these processes we analyzed DL1ICD-expressing male or homozygous female embryos by WISH using probes for Uncx4.1, Myogenin (Myog), Neurogenin1 (NeuroG1) as well as the Notch target Hey1. In addition, expression of Hes5 and Hey2, and of Nefm and Isl1 was analyzed by quantitative RT-PCR.

**Figure 4:**
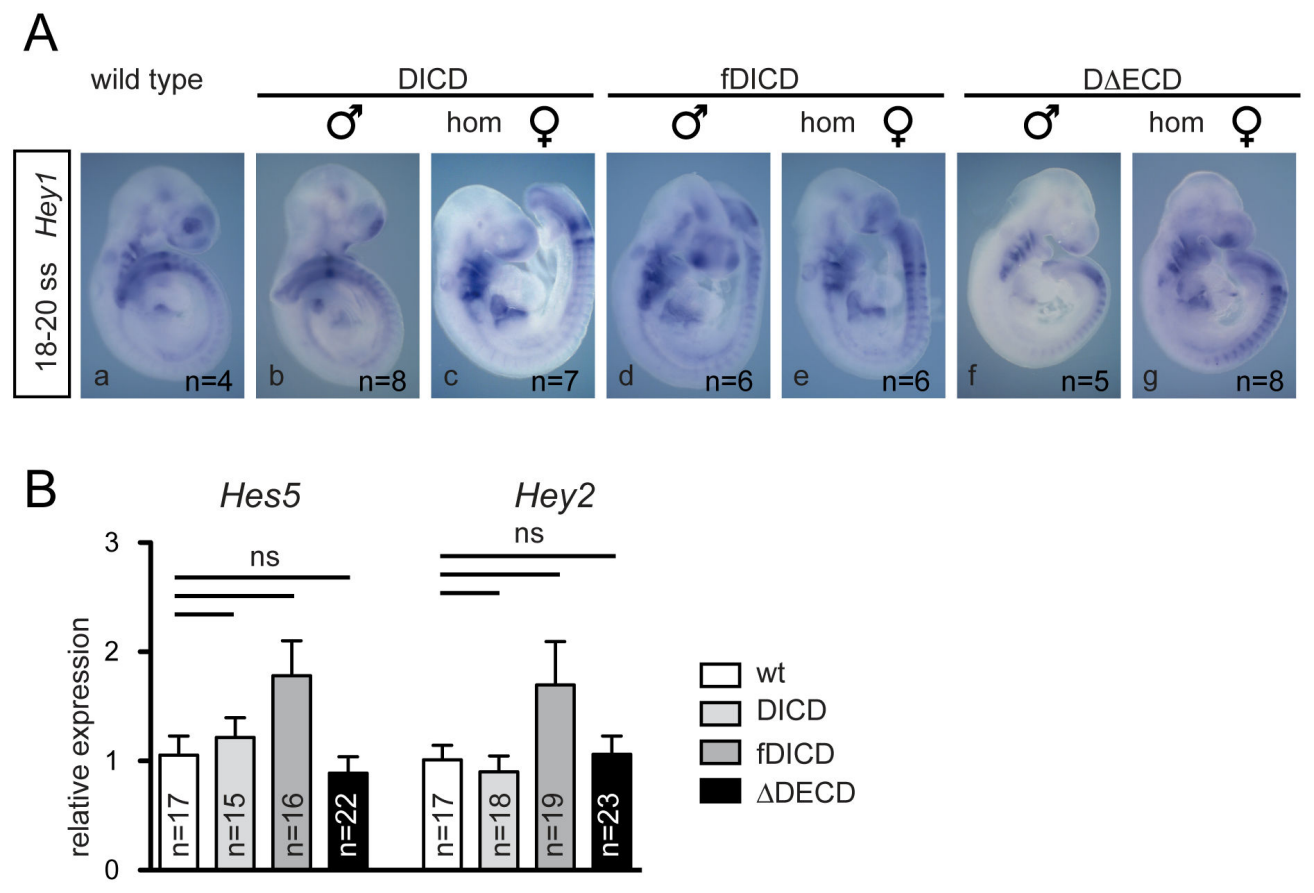
Normal Notch target gene expression in embryos expressing DL1ICD variants. (A) Whole mount in situ hybridization of wild type and DL1ICD-expressing embryos showing normal Hey1 expression. (B) qRT-PCR analysis of Hes5 and Hey2 expression in wild type and DL1ICD-expressing embryos. Indicated are means and SEM of expression levels determined in individual wild type and transgenic embryos. ns: not significant (p>0.05).

Disruption of Notch signaling causes loss or reduction of Uncx4.1 expression [28], which marks posterior somite compartments [42]. Interference of DL1ICD with Notch activity thus should affect the regular pattern of Uncx4.1. However, all analyzed transgenic embryos (≥3 male, and ≥3 homozygous female embryos expressing each of the DL1ICD variants) had Uncx4.1 expression patterns indistinguishable from wild type embryos (Figure 3C a-g). During muscle differentiation Notch activity is required to prevent premature and excessive muscle differentiation [38,43]. To analyze whether early muscle differentiation might be affected we analyzed Myog expression, which marks differentiating myoblasts in somites [44]. Myog expression was virtually identical in wt (n=3) and transgenic embryos (n≥4, respectively; Figure 3C h-n). To address whether DL1ICDs affect neuronal differentiation in vivo we analyzed expression of the Notch target NeuroG1, which is expressed in sensory neuron precursors [45]. As observed with the other markers, expression of NeuroG1 in DL1ICD embryos was indistinguishable from wild type (Figure 3D, and data not shown), suggesting that neurogenesis proceeds normally in DL1ICD embryos. Consistent with the in situ analyses the slight increases of the expression levels of Nefm and Isl1 detected by qRT-PCR were statistically not significant (p>0.05). Thus, our results are consistent with previous observations in frog embryos where over-expressed Xenopus DL1ICD had no effect on neurogenesis [24]. To detect potential effects on Notch signaling more directly we analyzed the Notch target Hey1 by in situ hybridization, and Hes5 and Hey2 by qRT-PCR. As with the other markers, Hey1 expression in DL1ICD expressing embryos (n≥5) was indistinguishable from wild type (Figure 4A), and only non-significant changes (p>0.05) in Hes5 and Hey2 expression levels were detected (Figure 4B). Thus, DL1ICD variants do not obviously interfere with transcription of Notch target genes in vivo, which is in line with the inability of DICD to affect expression from the Hes1 or Hes5 promoter in cultured cells [25]. 

### Ineffective nuclear translocation of mouse DICD in cultured cells

**Figure 5:**
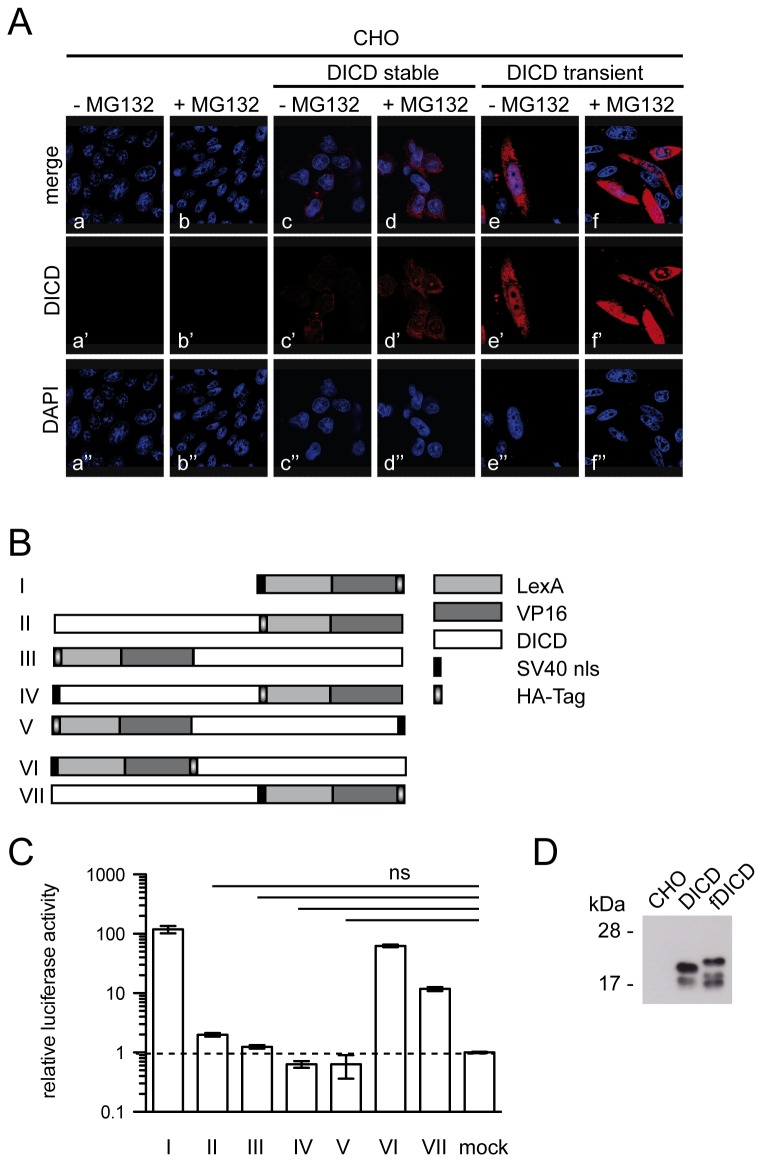
Inefficient nuclear translocation and cleavage of DICD. (A) Detection of DICD stably (c, d) or transiently (e, f) expressed in CHO cells in the absence (c, e) or presence (d, f) of the proteasome inhibitor MG132. (B) Schematic representation of DICD-LexAVP16 fusion constructs. (C) Activation of lexA operator driven luciferase in CHO cells. (D) Western blot of cell lysates of CHO cells stably expressing DICD and fDICD.

Potential functions of DICD have been suggested based on its nuclear localization [20-22,26]. However, we observed very little if any nuclear staining in cells that stably expressed DL1ICDs at low levels, in contrast to cells with high transient overexpression (Figure 5 A, and data not shown), raising the question how efficiently mouse DICD translocates to the nucleus. The human DL1ICD contains two nuclear localization signals (NLSs), each of which is sufficient for nuclear localization [27]. In the ICD of mouse DLL1 these NLSs both have one amino acid exchange, the functional consequence of which is unclear. To get a quantitative assay for the efficiency of nuclear localization of mouse DICD, we linked DICD to the lexA DNA binding domain fused to the transactivator domain of VP16 (LVP16, lacking a nuclear localization signal, Figure 5B) and measured the activation of a lexA operator-driven luciferase reporter stably integrated in the genome of CHO cells. As a positive control we used a construct without DICD, in which the SV40 NLS was fused to LVP16. Surprisingly, LVP16 fused either N-or C-terminally to DICD did not induce luciferase activity above background levels (Figure 5C), suggesting that the NLSs in mouse DICD do not function effectively. 

In CHO cells over-expressing DICD or fDICD smaller DICD fragments were detected in the absence of MG132 (Figure 5D), suggesting that DICD undergoes further proteolytical cleavage(s). To analyze this possibility further we added a known functional NLS at the N-terminus of DICD and LVP16 at the C-terminus and vice versa, or linked the NLS to LVP16 either at the N-or C-terminus (Figure 5B). If DICD is cleaved, constructs that contained the SV40 NLS at the N-terminus and LVP16 at the C-terminus of DICD and vice versa should show no or little activity due to separation of the NLS and LVP16 by cleavage. Conversely, when the NLS and LVP16 are present together at either the N-or C-terminus LVP16 should translocate to the nucleus and induce luciferase from the LexA operator driven promoter. Indeed, constructs that contained the SV40 NLS and LVP16 separated by DICD showed no significant activity. However, when the SV40 NLS and LVP16 were present together either at the N-or C-terminus of DICD luciferase expression from the LexA operator was induced (Figure 5). These findings support the idea that DICD is further cleaved in the cytoplasm, although formally we cannot rule out that the presence of DICD in LVP16 fusions interferes with the function of the SV40 NLS sequence when the NLS is separated from LVP16 by DICD.

In conclusion, the ubiquitous expression of three DICD variants at levels higher than endogenous DICD had no measurable effect on patterning processes that depend on DLL1/Notch signaling or expression of selected Notch target genes in vivo. This is in contrast to severe effects of expressing NICD in various tissues in transgenic mice [28,46,47]. Our in vivo results support recent in vitro data that showed that the inhibition of proteolysis of DLL1 in OP9 cells did not affect T-cell development [48], and that overexpression of various ligand ICDs had no significant effects on endothelial cell migration and sprouting angiogenesis in vitro [34]. Thus, our data do not support the existence of reverse signaling by cleavage of DLL1 under physiological conditions in vivo but rather support the notion that cleavage of mouse DLL1 is a means of inactivation and a prelude to further degradation as has been suggested also for Drosophila Delta [49]. 

## Materials and Methods

### Ethics Statement

Animal experiments were performed according to the German rules and regulations (Tierschutzgesetz), and approved by the ethics committee of Lower Saxony for care and use of laboratory animals LAVES (Niedersächsisches Landesamt für Verbraucherschutz und Lebensmittelsicherheit). Mice were housed in the central animal facility of Hannover Medical School (ZTL) and were maintained as approved by the responsible Veterinary Officer of the City of Hannover. Animal welfare was supervised and approved by the Institutional Animal Welfare Officer (Tierschutzbeauftragter). For embryo collection mice were sacrificed by cervical dislocation. The immunization of rats for the generation of monoclonal antibodies was approved by the Government of Upper Bavaria (Regierung von Oberbayern AZ 209.1/211-2531.6-4/99). Rats were housed in the animal facility of the Helmholtz-Zentrum München, and were maintained as approved by the responsible Veterinary Officer of the City of Munich. 

### Cell lines

E14TG2a ES cells were established from E14TG2a mice [50] that were kept on a mixed genetic background. ES-cells were cultured on gelatin-coated wells and in DMEM supplemented with 15% FCS, 100µM β-Mercaptoethanol, 1mM Sodium Pyruvate, 2mM Glutamax, 0,1mM non-essential Amino acids, 10000 U/ml Pen/Strep and LIF. CHO cells were cultured on tissue culture plates in DMEM/F12 supplemented with 10% FCS, 2mM Glutamax, and 10000 U/ml Pen/Strep.

### Generation of ES cells carrying mouse DL1ICD constructs in the HPRT locus

Expression constructs were introduced by homologous recombination into the genome of E14TG2a ES cells [50]. These cells carry an approximately 35 kb deletion at the HPRT locus and are sensitive to HAT selection. The targeting vector pMP8 [30] restores HPRT expression and HAT resistance in E14TG2a cells upon homologous recombination, thus allowing for direct selection of correctly targeted ES cells. Transgene constructs were introduced into pMP8 upstream of the Hprt locus by conventional cloning or InFusion cloning (Clontech; according to the manufacturer's instructions), linearized with Mfe1 and introduced into E14TG2a ES cells by electroporation. HAT resistant clones were expanded and the integrity of the insertion verified by PCR spanning the 5‘ and 3‘ homology arm, respectively. Recombination in ES cells was induced by electroporating a Cre expression vector into ES cells. Individual ES cell clones were expanded and analyzed for Cre-mediated recombination by PCR.

### Generation and genotyping of transgenic mice and embryos

Independently targeted ES cell clones obtained with each construct were used for chimera production. To activate transgene expression, germ line chimeras were crossed to ZP3::Cre mice and double heterozygous females bred to wild type CD1 males. Genomic DNA was isolated from tail biopsies of adult mice or from yolk sacs at different developmental stages. Recombined activated mouse DL1ICDs in the HPRT locus were genotyped by PCR using the following primer pairs: CRN4F (TGCTACCTGTTCATGCCTTCT) and FICD Rev 2 (CCCACAGGTTTCAGGTGGAGGCTGGTG). Wild type HPRT was detected using HPRT 5 del2F (TGGGCATTGGATCTCATTTTA) and HPRT 5 del2B (GATATCAAGCAGAGCCAGGAAG), the presence of the Y-chromosome using YMTFP1 (CTGGAGCTCTACAGTGATGA) and YMTRC2 (CAGTTACCAATCAACACATCAC) primers. 

### In situ hybridization

Embryos were collected, processed and hybridized to digoxigenin-labeled antisense probes under identical conditions by standard procedures [51]. 

### Antibodies

Anti-p21 WAF1/Cip1: clone CP74 (Millipore). Anti-HA: clone 3F10 (Roche). Anti-DLL1 antibodies: an affinity purified custom polyclonal rabbit anti-DLL1 ICD antibody directed against peptide C-RKRPESVYSTS (amino acids 688-695 of full length DLL1) in the intracellular domain of DLL1 (Biogenes), and a monoclonal antibody. The monoclonal antibody was generated against a peptide comprising amino acids _681_RGGEIPDRKRPESVY_695_ of mouse DLL1 (PSL, Heidelberg). Rats were immunized with a mixture of 50µg peptide-KLH, 5 nmol CPG oligonucleotide (Tib Molbiol, Berlin), 500µl PBS and 500µl IFAs. After a six-week interval a final boost without adjuvant was given three days before fusion of the rat spleen cells with the murine myeloma cell line P3X63-Ag8.653. Hybridoma supernatants were tested in ELISA using the specific peptide or an irrelevant peptide coupled to ovalbumin. RGG 2A5 of rat IgG2a subclass was used in this study.

### Western Blot analysis

Cells were lysed in 2x Sample Buffer for 3 minutes on ice and sonicated. In some experiments cells were treated with the proteasome inhibitor MG132 (Sigma-Aldrich, 25µM) for 8 hours or with γ-Secretase Inhibitor IX (Calbiochem; 10µM) for 4h prior to lysis. Single E10.5 embryos were lysed in 100µl 2x Sample Buffer on ice and sonicated. Proteins were separated by SDS-PAGE and blotted onto transfer membrane (Immobilon, Millipore). Membranes were blocked by 5% milk powder in PBST for 30 minutes and incubated with antibodies (anti-DLL1 peptide ab 1:500, 2A5 1:50, anti-HA 1:10000, anti p21 1:1000 in 5% dry milk powder in PBST) washed 3 times with PBST, incubated with horseradish peroxidase coupled secondary antibody ECL™ α-rat IgG or ECL^TM^ α-rabbit IgG or ECL^TM^ α-mouse-IgG (GE Healthcare; 1:10000, respectively) in 5% milk powder for 1h and washed 3 times in PBST. Bound antibodies were visualized using ECL Western Blotting Reagents (GE Healthcare) on Amersham Hyperfilm^TM^ ECL (GE Healthcare.

### Proliferation of ES-cells

200000 ES cells carrying the unrecombined or recombined DICD, DfICD or ΔDECD constructs were seeded per well of a 6 well plate coated with 0.1% gelatin. After 24 h, 48 h and 72 h, respectively, cells were trypsinized and the total cell numbers determined using a hemocytometer. All experiments were done in triplicates.

### RNA preparation from embryos and cDNA synthesis

E9.5 17-19 somite stage transgenic embryos (D ICD, fDICD Flag and DΔECD) were individually transferred into RNAlater (Ambion,#AM7020) and RNA was isolated using TriReagent (Sigma, #T9424) according to the manufacturer`s protocol. cDNA was synthesized from one quarter of each RNA preparation using the Thermoscript RT-PCR System (Invitrogen, #11146-016) according to the manufacturer`s protocol. 

### Quantitative real-time PCR

qRT-PCR was performed with Platinum TaqPCRx DNA Polymerase (Invitrogen, #11509-015) according to the basic protocol with SYBR Green and Rox (Invitrogen, #12223-012) in a 25µl reaction volume in a 7500 Fast Real-Time PCR System (Applied Biosystems) in duplicate. 1/10 and 1/50 of the synthesized cDNA from aggregates and embryos, respectively, was used for each reaction. Results were analyzed using 7500 Fast System SDS-Software (Applied Biosystems). Only measurements with a standard deviation of doublets less than 0.3 were considered further. Gene-specific primers selected for nearly 100% amplification efficiency were used to amplify short fragments. Primers used were: Nefm I1F1 (CGCCACAACCACGACCTCAG) and Nefm I1B1 (TCCCCGAAGTTCATTTTCCAAC); Isl1 I F1(CCTCTTACAGATATGGGAGACATGG) and Isl1 I5B1 (GCAACCAACACACAGGGAAA); Hes5 I5F2(TGGCGGTGGAGATGCTCAG) and Hes5 I1B1 (GCTGCTGTTGATGCGGTCC); Hey2 I4F1 (CTCCAGGCTACAGGGGGTAAAG) and Hey2 I4B2(CAAGCACTCTCGGAATCCAATG). β-actin expression was determined using β-Actin I4F1 (CTCTTTTCCAGCCTTCCTTCTT) and β-actin I4B2(GAGGTCTTTACGGATGTCAACG). β-actin levels were set to 1 and compared to the relative expression of the other genes. Ct-values were translated into fold change of expression levels using the ΔΔCt-method [52].

### Neuronal differentiation of ES cells

200000 ES cells, respectively, were cultured in medium containing LIF in hanging drops for 4 days to generate aggregates of defined size. Subsequently, to induce differentiation aggregates were cultured for 4 days in medium without LIF. During this period 10 nM retinoic acid was added to the positive controls. After 8 days 10 embryoid bodies of each cell line were pooled and transferred into one well of a 24-well plate coated with 0.1% gelatin, and cultured for additional 8 days. Cells were lysed, total RNA was isolated and used for cDNA synthesis using the Thermoscript RT-PCR System (Invitrogen, #11146-016) according to the manufacturer`s protocol. 

### Statistical analyses

Statistical analyses were done using Prism software (GraphPad). qRT-PCR results were analyzed by one-way ANOVA and expression levels of each analyzed gene compared between wild type and transgene-expressing cells or embryos using Dunnet's Multiple Comparison Test with a significance level of 0.05. Non-linear regression was used to fit counts of ES cells and to calculate doubling times. Luciferase measurements were analyzed by one-way ANOVA and activities obtained with each protein compared to empty vector control using Dunnet's Multiple Comparison Test with a significance level of 0.05.

### *lexA* activation assay

For measuring nuclear translocation of DICD the DNA binding domain of the bacterial LexA protein (residues 1-87[53,54]) linked to the transactivation domain of VP16 was fused to DICD. A synthetic LexA operator containing four operator sequences [55] was cloned into the luciferase reporter plasmid pGL4.27. CHO cells carrying the reporter were established and validated by PCR using following Primers: Seq LexOP 1Fwd 1 (GGCCTAACTGGCCGGTACCTG) and Seq LexOP Rev1 (AAGCTGGAAGTCGAGCTTCCATTA). Cells were transfected with DICD LexA-VP16 expression vectors and the Renilla luciferase expression vector pRL-TK (Promega). After 24 hours cells were lysed and luciferase activity determined using the Dual-Luciferase Reporter 1000 Assay system (Promega) according to the manufacturer`s instructions and a Veritas 9100-002 Microplate Luminometer (Turner Biosystems). Firefly luciferase activity was normalized to the activity of Renilla luciferase. The expression of DICD LexAVP16 proteins was verified by Western Blot analysis.
